# Regional disparities in Dementia-free Life Expectancy in Japan: An ecological study, using the Japanese long-term care insurance claims database

**DOI:** 10.1371/journal.pone.0280299

**Published:** 2023-05-25

**Authors:** Mikako Yoshikawa, Etsu Goto, Jung-ho Shin, Yuichi Imanaka

**Affiliations:** 1 Faculty of Medicine, Kyoto University, Kyoto, Japan; 2 Department of Healthcare Economics and Quality Management, Graduate School of Medicine, Kyoto University, Kyoto, Japan; Tehran University of Medical Sciences, ISLAMIC REPUBLIC OF IRAN

## Abstract

**Background:**

The number of people with dementia increases in an aging society; therefore, promoting policies for dementia throughout the community is crucial to creating a dementia-friendly society. Understanding the status of older adults with dementia in each region of Japan will be a helpful indicator. We calculated Dementia-free Life Expectancy and aimed to examine regional disparities and their associated factors.

**Methods:**

We calculated Dementia-free Life Expectancy and Life Expectancy with Dementia for each secondary medical area in Japan based on the Degree of Independence in Daily Living for the Demented Elderly, using data extracted from the Japanese long-term care insurance claims database. We then conducted a partial least squares regression analysis, the objective variables being Dementia-free Life Expectancy and Life Expectancy with Dementia for both sexes at age 65, and explanatory regional-level variables included demographic, socioeconomic, and healthcare resources variables.

**Results:**

The mean estimated regional-level Dementia-free Life Expectancy at age 65 was 17.33 years (95% confidence interval [CI] 17.27–17.38) for males and 20.05 years (95% CI 19.99–20.11) for females. Three latent components identified by partial least squares regression analysis represented urbanicity, socioeconomic conditions, and health services-related factors of the secondary medical areas. The second component explained the most variation in Dementia-free Life Expectancy of the three, indicating that higher socioeconomic status was associated with longer Dementia-free Life Expectancy.

**Conclusions:**

There were regional disparities in secondary medical area level Dementia-free Life Expectancy. Our results suggest that socioeconomic conditions are more related to Dementia-free Life Expectancy than urbanicity and health services-related factors.

## Introduction

Today, the world’s population is aging, especially in developed countries, and the number of people with dementia is increasing [1]. Japan is one of the most aged countries, and it has the highest prevalence of dementia (23.3 per 1,000 people) in the world [2], which burdens people with dementia, their communities, and the nation as a whole [3, 4]. With the growing importance of addressing the social problems of dementia, the Japanese government released the "Framework for Promoting Dementia Care" in 2019 [5]. It aims to delay the onset of dementia and create a society where people can live their daily lives with hope, regardless of whether they have dementia. This framework also mentions the need to collect and analyze evidence of effective measures to promote at the community level.

Many studies have reported various healthy life expectancies to assess a population’s health status [6, 7]. For example, dementia-free life expectancy has been calculated in several countries via different methods, revealing that a healthy lifestyle is associated with a larger proportion of remaining years without dementia [8–11]. Those studies calculated dementia-free life expectancy by predicting the country’s prevalence from extracted data, but few have clarified differences among smaller units or regions within a country [12].

Under Japan’s Long-term care insurance (LTCI) system, people aged 65 years or older and persons aged 40 to 65 years with the specified conditions receive a "long-term care need certification" to utilize LTCI services such as home-visit care and daycare services. The level of need for long-term care is determined through a computerized primary judgment and a secondary judgment by a certification committee. Service applicants are then entitled to receive LTCI services according to their level of need. One of the criteria for assessing the level of need for LTCI services is the "Degree of Independence in Daily Living for the Demented Elderly," which evaluates the level of independence considering their dementia-related symptoms and behaviors. We can extract this information from the Japanese long-term care insurance claims database, which covers LTCI users all over Japan, therefore allowing us to make nationwide comparisons among regions in Japan.

This study aimed to calculate and explore the regional difference in Dementia-free Life Expectancy (De-FLE) based on the Degree of Independence in Daily Life for the Demented Elderly. These metrics will enable us to explore whether regional disparities exist in the dementia-related independence period and what factors are associated with these trends. We expect this study to help increase De-FLE and reduce regional disparities.

## Method

### Data source

We extracted cases from the Japanese long-term care insurance claims database of the Ministry of Health, Labour and Welfare (MHLW) by the Degree of Independence in Daily Living for the Demented Elderly. This database includes information on long-term care certifications and long-term care receipts collected nationwide. We also aggregated regional variables from publicly available data sources, shown in Table 1.

**Table 1:** Factors related to regional characteristics, denominators used in calculations, and data sources.

	Variables	Denominator	Sources
Demographic variables	Total population [100,000person]		Population Census 2015
	Population density [person/ha]	Inhabitable area[ha]	Population Census 2015
	Proportion of women in the working-age population [%]	100 working-age people	Population Census 2015
	Proportion of child population [%] (under 15)	100 people	Population Census 2015
	Proportion of elderly population [%] (65 and over)	100 people	Population Census 2015
	Proportion of foreign population [%]	100 people	Population Census 2015
	Mean household size [person]	100 private household members	Population Census 2015
	Proportion of single-mother households [%]	100 Nuclear families	Population Census 2015
	Proportion of population aged 75 and over [%]	100 people	Population Census 2015
	Proportion of single households [%]	100 private households	Population Census 2015
	Proportion of unmarried people [%]	100 people aged 15 and more	Population Census 2015
	Proportion of older single households [%]	100 people aged 65 and more	Population Census 2015
	Social increase/decrease per total population [%]	100 people	Annual report on internal migration in Japan derived from the basic resident registration 2015
	Natural increase/decrease per population [%]	100 people	Annual report on internal migration in Japan derived from the basic resident registration 2015
Socioeconomic variables	Unemployment rate [%]	100 people in labor force	Population Census 2015
	Proportion of elderly in employment [%]	100 people aged 65 and more	Population Census 2015
	Proportion of persons employed in primary industry [%]	100 employed persons	Population Census 2015
	Proportion of persons employed in secondary industry [%]	100 employed persons	Population Census 2015
	Proportion of persons employed in tertiary industry [%]	100 employed persons	Population Census 2015
	Proportion of persons working in the same municipalities [%]	100 employed persons	Population Census 2015
	Proportion of elementary school and lower secondary school graduates [%]	100 total graduates	Population Census 2010
	Proportion of high school graduates [%]	100 total graduates	Population Census 2010
	Proportion of junior college or university graduates [%]	100 total graduates	Population Census 2010
	Commuters by railway or train [%]	100 commuters going to work and to school	Population Census 2010
	Commuters by private automobiles [%]	100 commuters going to work and to school	Population Census 2010
	Number of suicides per population [person]	10,0000 people	Suicide Statistics (by death date & place of residence) 2017
	Number of administrative officers [person]	10,000 people	Survey on the Total Number Management of Civil Servants in Local Governments 2017
	Municipal resident taxes revenue [1,000yen]	100 people aged 15 and more	Annual Statistical Report on Local Government Finance 2017
	Tobacco taxes revenue [1,000yen]	100 people aged 15 and more	Annual Statistical Report on Local Government Finance 2017
	Financial power index [–]	Base fiscal revenue / Base fiscal demand	Annual Statistical Report on Local Government Finance 2018
	Proportion of people applied national pension contribution exemption [%]	100 people insured only by the National Pension	Survey on Public Pension Enrollment 2017
	Pension benefit per Employees’ pensioner [1,000yen]	employee pension recipients	Survey on Public Pension Enrollment 2017
	Proportion of users of long-term care insurance services who pay their own taxes [%]	100 people insured for long-term care insurance	Fact-finding Survey on Project of Long-term Care 2017
	Number of dementia supporters [person]	100,000 people aged 65 and more	Dementia Supporter Caravan Statistics 2017
Variables representing medical and nursing resources	Number of hospitals per 10,0000 people	100,000 people	Survey of Medical Institutions, Hospital Report 2017
	Number of general clinics per 10,000 people	100,000 people	Survey of Medical Institutions, Hospital Report 2017
	Number of dental clinics per 10,000 people	100,000 people	Survey of Medical Institutions, Hospital Report 2017
	Number of hospital beds per 1,000 people	1,000 people	Survey of Medical Institutions, Hospital Report 2017
	Number of hospitals per 1ha	Inhabitable area[ha]	Survey of Medical Institutions, Hospital Report 2017
	Number of general clinics per 1ha	Inhabitable area[ha]	Survey of Medical Institutions, Hospital Report 2017
	Number of physicians [person]	100,000 people	Statistics of Physicians, Dentists and Pharmacists 2018
	Number of dentists [person]	100,000 people	Statistics of Physicians, Dentists and Pharmacists 2018
	Number of pharmacists [person]	100,000 people	Statistics of Physicians, Dentists and Pharmacists 2018
	Number of welfare institutions for the aged	100,000 people aged 65 and more	Survey of Social Welfare Institutions 2017
	Number of long-term care health facilities	100,000 people aged 65 and more	Survey of Institutions and Establishments for Long-term Care 2017
	Capacity of long-term care health facilities [person]	1,000 people aged 65 and more	Survey of Institutions and Establishments for Long-term Care 2017
	Number of elderly nursing facilities	100,000 people aged 65 and more	Survey of Institutions and Establishments for Long-term Care 2017
	Capacity of elderly nursing facilities [person]	1,000 people aged 65 and more	Survey of Institutions and Establishments for Long-term Care 2017
	Number of child welfare institutions	100,00 people under 15	Survey of Social Welfare Institutions 2017
	Number of institutions for support of the disabled	100,000 people	Survey of Social Welfare Institutions 2017
	Proportion of in-home services users [%]	100 nursing care insurance users	Fact-finding Survey on Project of Long-term Care 2017
	Proportion of welfare institutions services users [%]	100 nursing care insurance users	Fact-finding Survey on Project of Long-term Care 2017
	Proportion of community-based services users [%]	100 nursing care insurance users	Fact-finding Survey on Project of Long-term Care 2017

### Geographical units

Secondary medical areas (SMAs), which consist of several municipalities, were used as the unit of this analysis. Japan’s Medical Care Act stipulates SMAs are the units that take comprehensive measures to foster cooperation between health care, medical care, and welfare. There are 335 SMAs in Japan, with a mean population of about 4 million in each SMA. We aggregated public statistical data by SMA and utilized it in the analysis. We used data in 2017, but data submission from long-term care insurers became mandatory after 2018. In 2017, some insurers did not submit data or had significantly fewer data. To omit those insurers, we checked the list of long-term care insurers against the specific extraction data and identified 334 SMAs. We excluded one SMA with missing data from the 2015 census owing to the Great East Japan Earthquake and included 333 SMAs in the analysis.

### Dementia-free Life Expectancy

In our study, De-FLE refers to the period before dementia symptoms become severe enough to require support from others. We also defined Life Expectancy (LE) with Dementia as the period in which people receive long-term care due to cognitive difficulties, representing the period between life expectancy and De-FLE. To calculate these metrics, we utilized the "Degree of Independence in Daily Living for the Demented Elderly," which was created by the MHLW to facilitate objective and quick assessments by public health nurses, nurses, social workers, and care workers [13]. When the elderly in need apply for LTCI certification, investigators designated by the municipality visit their place of residence and judge their level of independence. Their family physicians also use the criteria to evaluate their independence level. The Degree of Independence in Daily Living for the Demented Elderly classifies applicants into five categories regarding their communication capacity, symptoms, and behaviors (S1 Table). In this study, we defined "unhealthy" as Independence Level II or higher to calculate De-FLE. Those assessed as Independence Level II usually live at home, but since it is challenging for them to live alone as they need support from the people around them, they often use home services in the daytime.

We extracted data from the Japanese long-term care insurance claims database in 2017 provided by the MHLW and calculated De-FLE using the number of independent degrees of daily living for the demented elderly. We defined LE with Dementia as the period between life expectancy and healthy life expectancy. The calculation followed the "Healthy Life Expectancy Calculation Program" published by the MHLW research group [14].

### Statistical analysis

We conducted a partial least squares (PLS) regression with SMA-level De-FLE and LE with Dementia for both sexes at age 65 in 2017 as the objective variables and regional factors as explanatory variables. We selected explanatory variables based on previous studies and the World Health Organization Guidelines on Dementia: "Risk Factors for Dementia," and then aggregated them by SMAs, as shown in Table 1 [15].

PLS regression analysis is commonly used in econometrics, and in recent years, a few public health studies have adopted it [16, 17]. This model first extracts the latent components to have the largest covariance with the objective variables, then uses them to perform regression analysis. It can deal with multicollinearity among variables, making it useful for cases with many correlated explanatory variables [18]. We identified the number of latent factors as follows. First, we used leave-one-out cross-validation to identify the number of factors that minimizes the predicted residual sum of squares (PRESS) by this process [19]. The model with the number of latent factors identified here does not often show significant differences from a model with fewer factors. We performed Van der Voet’s test to test whether the difference was significant. Van der Voet’s test is a statistical test that compares the predicted residuals of different models [20]. We finally identified the least number of latent factors among the models that none were significantly different from the model with the smallest PRESS. All statistical analyses were conducted using JMP Pro 16.1 (SAS Institute Inc., Cary, NC).

This study was conducted in accordance with Ethical Guidelines for Medical and Biological Research Involving Human Subjects and the study was approved by the Ethics Committee, Kyoto University Graduate School and Faculty of Medicine (approval number: R0438) with a waiver of informed consent prior to data collection.

## Results

### Descriptive statistics for SMA-level LE, De-FLE, LE with Dementia, and regional variables

The mean SMA-level De-FLE at age 65 in 2017 was 17.33 years (95% confidence interval [CI] 17.27–17.38 years) for males and 20.05 years (95% CI 19.99–20.11 years) for females, a difference of 2.72 years, with standard deviations of 0.56 years and 0.54 years, respectively (Table 2).

**Table 2:** Descriptive statistics for SMA-level LE, De-FLE, LE with Dementia, and regional variables (n = 333).

Explanatory Variables					percentile			
	VIP^a^^,^^b^	Mean		SD^a^		25%	50%	75%
Total population [100,000person]	✓	38.133		45.301		10.190	22.723	47.676
Population density [person/ha]		16.625		27.584		3.678	7.207	13.793
Proportion of women in the working-age population [%]	✓	49.607		1.288		48.797	49.555	50.491
Proportion of child population [%] (under 15)	✓	12.302		1.510		11.506	12.288	13.174
Proportion of elderly population [%] (65 and over)	✓	29.787		5.146		26.126	29.457	33.019
Proportion of foreign population [%]	✓	0.956		0.763		0.437	0.708	1.247
Mean household size [person]	✓	2.449		0.230		2.286	2.464	2.602
Proportion of single-mother households [%]	✓	2.710		0.626		2.277	2.649	3.057
Proportion of population aged 75 and over [%]	✓	50.674		4.506		47.325	50.935	54.106
Proportion of single households [%]	✓	30.489		5.871		26.299	29.779	33.633
Proportion of unmarried people [%]		24.718		3.296		22.553	24.319	26.670
Proportion of older single households [%]	✓	16.598		4.152		13.460	15.939	19.655
Social increase/decrease per total population [%]		-0.259		0.411		-0.521	-0.284	-0.030
Natural increase/decrease per population [%]	✓	-0.613		0.408		-0.892	-0.605	-0.324
Unemployment rate [%]	✓	4.143		0.809		3.552	4.110	4.599
Proportion of elderly in employment [%]	✓	23.290		3.429		21.121	23.088	25.325
Proportion of persons employed in primary industry [%]	✓	7.715		6.294		2.690	5.970	11.827
Proportion of persons employed in secondary industry [%]	✓	24.784		6.916		19.508	23.986	29.333
Proportion of persons employed in tertiary industry [%]		64.170		6.408		59.286	63.663	68.985
Proportion of persons working in the same municipalities [%]	✓	66.129		17.910		53.754	69.513	80.123
Proportion of elementary school and lower secondary school graduates [%]		22.415		8.611		16.239	21.741	28.269
Proportion of high school graduates [%]		44.893		6.383		41.655	45.937	49.373
Proportion of junior college or university graduates [%]	✓	24.846		7.352		18.914	23.905	29.254
Commuters by railway or train [%]	✓	7.363		10.421		1.729	3.007	7.054
Commuters by private automobiles [%]		61.128		19.075		57.210	67.846	74.077
Number of suicides per population [person]	✓	1.811		0.541		1.483	1.712	2.012
Number of administrative officers [person]	✓	67.101		24.772		49.509	59.661	78.244
Municipal resident taxes revenue [1,000yen]		55.063		18.063		43.576	52.372	61.985
Tobacco taxes revenue [1,000yen]	✓	7.846		1.462		7.002	7.646	8.334
Financial power index [–]	✓	0.591		0.233		0.403	0.569	0.771
Proportion of people applied national pension contribution exemption [%]	✓	20.787		6.064		16.070	19.923	24.579
Pension benefit per Employees’ pensioner [1,000yen]	✓	588.488		101.853		515.039	595.649	667.921
Proportion of users of long-term care insurance services who pay their own taxes [%]	✓	37.717		5.340		33.9	38	41.7
Number of dementia supporters [person]	✓	0.200		0.105		0.133	0.178	0.233
Number of hospitals per 10,0000 people	✓	8.236		3.943		5.313	7.332	10.373
Number of general clinics per 10,000 people	✓	77.428		19.333		65.459	76.071	86.775
Number of dental clinics per 10,000 people	✓	47.953		12.947		41.317	46.878	51.546
Number of hospital beds per 1,000 people	✓	13.818		4.779		10.557	13.062	16.360
Number of hospitals per 1ha		0.991		1.292		0.308	0.565	1.035
Number of general clinics per 1ha		14.266		31.779		2.938	5.226	11.006
Number of physicians [person]	✓	221.173		100.266		166.757	197.310	246.103
Number of dentists [person]		69.170		30.096		56.778	63.988	72.553
Number of pharmacists [person]		201.299		104.249		159.553	185.476	229.036
Number of welfare institutions for the aged		20.167		9.819		13.327	18.837	24.357
Number of long-term care health facilities		15.110		5.572		11.447	14.376	18.159
Capacity of long-term care health facilities [person]		12.141		3.311		9.813	12.086	14.232
Number of elderly nursing facilities	✓	29.612		10.369		21.936	28.423	35.391
Capacity of elderly nursing facilities [person]		18.654		5.584		14.974	17.807	20.954
Number of child welfare institutions		3.293		1.234		2.383	3.022	3.960
Number of institutions for support of the disabled		6.482		4.048		3.778	5.593	8.192
Proportion of in-home services users [%]		16.896		3.466		14.317	16.821	19.338
Proportion of welfare institutions services users [%]	✓	57.603		5.233		54.688	58.255	61.015
Proportion of community-based services users [%]	✓	13.572		2.974		11.630	13.284	15.494
Objective variables [year]								
LE at 65 years old (Female)		24.093		0.541		22.234	23.779	24.073
De-FLE at 65 years old (Female)		20.054		0.557		18.450	19.711	20.014
LE with Dementia at 65 years old (Female)		4.039		0.483		2.591	3.725	4.060
LE at 65 years old (Male)		19.207		0.560		17.322	18.892	19.194
De-FLE at 65 years old (Male)		17.329		0.538		15.520	17.014	17.296
LE with Dementia at 65 years old (Male)		1.878		0.277		1.175	1.698	1.869

^a^ VIP, the variable importance in projection; SD, Standard deviation

^b^ Of the 53 variables aggregated as SMA-level explanatory variables, there were 35 with a VIP 0.8 or higher in the initial PLS regression analysis adopted as explanatory variables in this paper as indicated by ✓.

### PLS regression analysis

Cross-validation results showed that the PRESS was minimal when 15 factors were extracted. Then, three components were finally identified based on the outcome of Van der Vote’s Test. Factor loadings and the variable importance in projection (VIP) for each of the three factors are shown in Table 3.

**Table 3:** Each component’s factor loadings/correlations and variable importance in the projection.

	Component 1	Component 2	Component 3	VIP^a^
Coefficient of determination of explanatory variables	0.312	0.123	0.095	
Coefficient of determination of objective variables	0.033	0.067	0.066	
De-FLE at 65 years old (Female)	-0.105	0.201	0.265	
LE with Dementia at 65 years old (Female)	0.141	-0.110	0.253	
De-FLE at 65 years old (Male)	0.233	0.458	0.232	
LE with Dementia at 65 years old (Male)	0.215	-0.079	0.276	
Proportion of junior college or university graduates [%]	0.924	0.008	0.045	0.938
Natural increase/decrease per population [%]	0.864	-0.163	-0.039	0.906
Proportion of users of long-term care insurance services who pay their own taxes [%]	0.841	0.271	-0.210	0.807
Financial power index [–]	0.832	-0.034	-0.373	0.930
Pension benefit per Employees’ pensioner [1,000yen]	0.827	0.120	-0.248	0.824
Proportion of commuters by railway or train [%]	0.720	-0.182	-0.018	0.805
Total population [100,000person]	0.688	-0.317	-0.057	0.927
Proportion of foreign population [%]	0.617	-0.008	-0.183	0.713
Number of dental clinics per 10,000 people	0.456	-0.397	0.312	0.942
Number of physicians [person]	0.455	-0.285	0.479	1.002
Proportion of child population [%]	0.448	0.024	0.019	0.771
Proportion of single households [%]	0.304	-0.650	0.386	1.215
Number of general clinics per 10,000 people	0.189	-0.149	0.573	0.862
Proportion of persons employed in secondary industry [%]	0.036	0.504	-0.580	1.180
Proportion of older single households [%]	0.027	-0.689	0.448	1.167
Proportion of women in the working-age population [%]	0.022	-0.206	0.409	0.903
Tobacco taxes revenue [1,000yen]	-0.077	-0.568	0.034	1.310
Proportion of elderly in employment [%]	-0.098	0.475	0.177	0.722
Number of dementia supporters [person]	-0.110	0.392	0.276	0.949
Unemployment rate [%]	-0.122	-0.662	-0.248	1.505
Proportion of community-based services users [%]	-0.147	0.207	0.381	0.931
Mean household size [person]	-0.163	0.651	-0.387	1.086
Proportion of single-mother households [%]	-0.360	-0.567	0.162	1.068
Number of suicides per population [person]	-0.429	-0.093	-0.093	1.217
Number of hospital beds per 1,000 people	-0.444	-0.202	0.432	0.860
Capacity of elderly nursing facilities [person]	-0.490	0.232	0.007	0.971
Number of welfare institutions for the aged	-0.502	0.169	0.394	0.884
Number of hospitals per 10,0000 people	-0.574	-0.152	0.417	1.007
Proportion of in-home services users [%]	-0.631	0.416	-0.108	0.921
Proportion of people applied national pension contribution exemption [%]	-0.655	-0.485	0.246	1.085
Number of administrative officers [person]	-0.670	0.098	0.311	1.080
Proportion of persons working in the same municipalities [%]	-0.724	0.010	0.270	1.022
Proportion of persons employed in primary industry [%]	-0.771	0.119	0.292	0.839
Proportion of population aged 75 and over [%]	-0.792	0.139	0.401	1.111
Proportion of elderly population [%]	-0.835	0.198	0.162	1.049
Proportion of elderly population [%]	-0.835	0.198	0.162	1.049

^a^ VIP, the variable importance in projection

The factor loadings in Table 3 show that Component 1 was positively loaded by the proportion of junior college or university graduates, the proportion of commuters traveling by railway or train, and the financial power index. In contrast, Component 1 was negatively loaded by the proportion of persons employed in primary industry and the proportion of elderly in the population. Thus, we named this component urbanicity. Component 2 was positively loaded by the proportion of persons employed in secondary industry and mean household size, while it was negatively loaded by the proportion of single households, tobacco taxes revenue per population, unemployment rate, and the proportion of single-mother households. We defined this as the socioeconomic conditions-related component. Component 3 was characterized by positive loadings on the number of general clinics per 100,000 people and the number of hospital beds per 1,000 people, and thereby named the healthcare resources component. The high score of each component represented the following variables: Component 1, high urbanization; Component 2, good socioeconomic conditions; and Component 3, rich healthcare resources.

Of the three components in Table 3, Component 1 had the largest coefficient of determination for the explanatory variable (31.2%), and Component 2 had the largest coefficient of determination for the objective variable (6.67%), which meant that Component 2 explained De-FLE the best of the three components.

**Fig 1:**
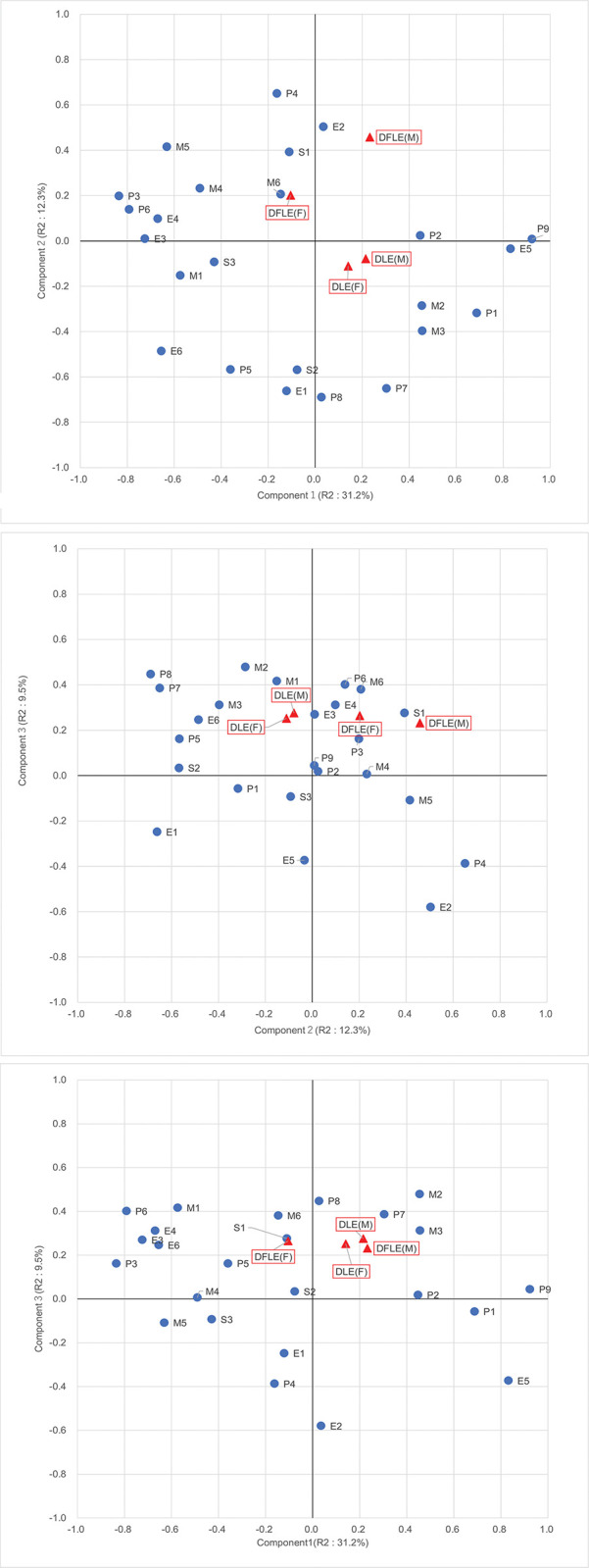
Correlation and loading plots for the first three components. (A) Component 1 and Component 2; (B) Component 2 and Component 3; (C) Component 1 and Component 3. Red triangles indicate the loadings of objective variables, and blue circles indicate the loadings of the explanatory variables. P1, Total population; P2, Proportion of child population; P3, Proportion of elderly population; P4, Mean household size; P5, Proportion of single-mother households; P6, Proportion of population aged 75 and over; P7, Proportion of single households; P8, Proportion of older single households; P9, Proportion of junior college or university graduates; S1, Number of dementia supporters per elderly population; S2, Tobacco taxes revenue per population; S3, Number of Suicides per population; E1, Unemployment rate; E2, Proportion of persons employed in secondary industry; E3, Proportion of persons working in the same municipalities; E4, Number of administrative officers; E5, Financial power index; E6, Proportion of people applied national pension contribution exemption; M1, Number of hospitals per 100,000 people; M2, Number of physicians; M3, Number of dental clinics per 100,000 people; M4, Capacity of elderly nursing facilities; M5, Proportion of welfare institutions services users; M6, Proportion of community-based services users; DFLE(F), De-FLE (Female); DLE (F), LE with Dementia (Female); DFLE(M), De-FLE (Male); DLE(M), LE with Dementia (Male).

Fig 1(A) shows the correlation and loading plot of variables for Components 1 and 2. De-FLE for males and LE with Dementia for both sexes were positively correlated with the score of Component 1. De-FLE for both sexes was positively correlated with the score of Component 2, especially for males. Some variables with high negative loadings with Component 2 had nearly zero loading on Component 1 (e.g., tobacco revenue per population and unemployment rate). Fig 1(B) and 1(C) show the correlation and loading plots of Components 2/3 and 1/3, respectively. All objective variables were positively correlated with Component 3. The variation in objective variables of Component 2 was much wider than that of Components 1 and 3.

## Discussion

In this study, we calculated SMA-level De-FLE and LE with Dementia for both sexes and then conducted PLS regression analysis to examine regional disparities in Japan. De-FLE and LE with Dementia varied among SMAs, and the mean SMA-level De-FLE was longer for women than men. Three latent components were identified, which represented urbanicity, socioeconomic conditions, and healthcare resources. Socioeconomic conditions explained De-FLE the best.

Component 1 explained the region’s characteristics well, but its contribution to De-FLE can be described as small based on the coefficient of determination. The score of Component 2 had a positive correlation with De-FLE. Therefore, De-FLE will be longer for persons living in better socioeconomic conditions. Component 2 can be referred to as the most crucial component in explaining De-FLE because it had the largest coefficient of determination for the objective variables of all three components. This result was consistent with the well-known fact that maintaining good socioeconomic status is essential to staying healthy [21]. In addition, an Improvement in living conditions will prolong De-FLE. The score of Component 3 was positively correlated with De-FLE, suggesting that better access to healthcare resources was also positively associated with longer De-FLE. The development of a healthcare delivery system may result in longer De-FLE [22, 23].

The score of Component 1 was positively correlated with LE with Dementia for both sexes. Several studies in the US have reported higher mortality among people living in non-metropolitan than in metropolitan areas [24, 25]. Component 2 had a negative and weak correlation with LE with Dementia, contrary to De-FLE. Socioeconomic conditions may affect De-FLE more than LE with Dementia. The score of Component 3 was positively correlated with LE with Dementia, suggesting that access to healthcare resources was also positively associated with these metrics. A large number of people with dementia might bring about the need to develop a healthcare delivery system, thereby leading to an increase in physicians. However, a causal relationship remains unclear, and further research is warranted.

As discussed above, socioeconomic conditions best explain the variation in De-FLE, compared with urbanicity and healthcare resources. Most of the variables that illustrate Component 2 well had little loadings on Component 1, as shown in Fig 1. For example, variables with high negative loadings on Component 2 were the unemployment rate, the proportion of single households, and tobacco taxes revenue per population. These variables had little loadings on Component 1, meaning that these variables were negatively associated with De-FLE regardless of urbanicity. On the other hand, variables with high positive loadings on Component 2 were the mean household size and the number of dementia supporters per elderly person in the population, implying that they were also positively associated with De-FLE. These also had little loadings on Component 1, which means that they are positively associated with De-FLE in both urban and rural areas. We will further discuss socioeconomic conditions below.

The unemployment rate was a critical variable of Component 2. A previous study suggested a significant relationship between work and self-rated health among older adults [26]. A sense of fulfillment is also necessary because working only for financial gain diminishes the health benefits of working after retirement [27]. Measures to reduce the unemployment rate by sufficiently arranging rewarding jobs might work toward extending De-FLE.

Tobacco taxes revenue per population was also a crucial variable of socioeconomic conditions. The adverse effects of tobacco on overall health, not just dementia, have led to the enforcement of many tobacco control and policy measures [28, 29]. Social factors such as poor housing conditions, low wages, isolated parents, unemployment, and homelessness are closely linked to high rates of smoking and low rates of smoking cessation, and smoking is a significant cause of illness and premature death [30]. Given the above report and our study, smoking will not only directly but indirectly harm a person’s health status and De-FLE.

The number of dementia supporters per elderly person in the population also explains the characteristics of socioeconomic conditions. Previous studies revealed that extensive community resources contribute to longer healthy life expectancy [31, 32]. Improved initiatives, such as introducing dementia supporters, may play a critical role in encouraging government and community residents to better understand people with dementia, which will help prevent isolation among the elderly [33].

Variables representing family structure, such as the proportion of older single households and mean household size, were influential factors in explaining socioeconomic conditions. Previous research has found that household size was negatively associated with mortality from dementia [34]. Another previous study noted that people who live alone are more likely to move into a nursing home than those who live with a family member [35]. Our study suggests that household size may also be negatively associated with the number of people needing care due to dementia. However, it is estimated that the proportion of single households will continue to increase, accounting for 40% of all households by 2040 [36]. Given this social trend, promoting the development of community-based support systems will be imperative to avoid burdening family members and ensure that everyone receives appropriate care.

As for gender differences, the correlation between Component 1 and De-FLE was positive for males and nearly zero for females, which means urbanicity could affect males and females differently. Previous studies on the association between city size and health showed that areas with higher urbanicity had more people aged 100 years and over and a lower risk of physical disability [37–39]. In addition, Component 2, which represents socioeconomic conditions, correlated with De-FLE better for males than females. Several reports have found gender differences in the association between socioeconomic conditions and healthy life expectancy [40, 41]. This study found that socioeconomic conditions explain De-FLE particularly well for males, suggesting the need for community support considering gender.

### Strengths and limitations

We examined regional disparities in the time before the elderly needed substantial long-term care. Using LTCI data and official statistical data allowed us to make comparative observations not only for specific regions but also across all regions of Japan. On the other hand, there are some limitations to this study. First, an ecological fallacy, a phenomenon in which observed associations true at the population level do not apply at the individual level, might occur in this study. We considered variables reported to associate with individual-level health status in previous studies and found consistent results. In addition, this study aimed to identify factors associated with regional differences in De-FLE rather than to explore individual-level factors. Furthermore, regional variables, such as the proportion of unmarried people and educational background, may not fully apply to the population over 65. Therefore, we used such regional variables to measure a neighborhood environment rather than directly describing the nature of the older population. Finally, some official statistical data, such as educational background and commuting patterns, were collected from the 2010 Census results, and the situation may have changed since that time. Continuous monitoring with new data is warranted.

## Conclusions

This study calculated Dementia-free Life Expectancy at the secondary medical area level as a new indicator for understanding the regional statuses of the elderly with dementia across the entire country of Japan and explored its association with regional variables. We found that socioeconomic conditions correlate with Dementia-free Life Expectancy more than urbanicity and healthcare resources factors. In order to achieve the extension of dementia-free life expectancy and the elimination of its regional disparities, it may be effective for the national and local governments to offer more comprehensive socioeconomic support and make efforts to gain the understanding and cooperation of local communities.

## Supporting information

S1 Table Degree of Independence in Daily Living for the Demented Elderly. (DOCX)
