# Supplementary material for: Regional disparities in Dementia-free Life Expectancy in Japan: An ecological study, using the Japanese long-term care insurance claims database
Source: PLoS One. 2023 May 25;18(5):e0280299. doi: 10.1371/journal.pone.0280299 (PMC10212174; doi:10.1371/journal.pone.0280299)
Supplement: S1 Table — (DOCX) [file pone.0280299.s001.docx]

Supplementary Table 1. Degree of Independence in Daily Living for the Demented Elderly

| Subjects are ranked into five categories, based on the level of communication capacity, symptoms, and behaviors. |
| --- |
| 1. Being with dementia but independent in daily life both in home and socially. |
| 2. Having occasional symptoms, behaviors or communication difficulties that interfere with daily life but independent if someone pays attention to them. |
| 3. Having some symptoms, behaviors or communication difficulties that interfere with daily life and requiring nursing care. |
| 4. Having frequent symptoms, behaviors or communication difficulties that interfere with daily life and requiring constant nursing care. |
| 5. Having significant psychiatric symptoms, problematic behaviors or serious physical illness and requiring specialized medical care. |
